# A comparison of clinical development pathways to advance tuberculosis regimen development

**DOI:** 10.1186/s12879-022-07846-w

**Published:** 2022-12-09

**Authors:** V. Chang, P. P. J. Phillips, M. Z. Imperial, P. Nahid, R. M. Savic

**Affiliations:** 1 Department of Bioengineering and Therapeutic Sciences, University of California San Francisco, San Francisco, CA USA; 2 UCSF Center for Tuberculosis, University of California San Francisco, San Francisco, CA USA

**Keywords:** Adaptive Clinical Trials, Tuberculosis, Clinical Trial Design

## Abstract

**Background:**

Current tuberculosis (TB) regimen development pathways are slow and in urgent need of innovation. We investigated novel phase IIc and seamless phase II/III trials utilizing multi-arm multi-stage and Bayesian response adaptive randomization trial designs to select promising combination regimens in a platform adaptive trial.

**Methods:**

Clinical trial simulation tools were built using predictive and validated parametric survival models of time to culture conversion (intermediate endpoint) and time to TB-related unfavorable outcome (final endpoint). This integrative clinical trial simulation tool was used to explore and optimize design parameters for aforementioned trial designs.

**Results:**

Both multi-arm multi-stage and Bayesian response adaptive randomization designs were able to reliably graduate desirable regimens in ≥ 95% of trial simulations and reliably stop suboptimal regimens in ≥ 90% of trial simulations. Overall, adaptive phase IIc designs reduced patient enrollment by 17% and 25% with multi-arm multi-stage and Bayesian response adaptive randomization designs respectively compared to the conventional sequential approach, while seamless designs reduced study duration by 2.6 and 3.5 years respectively (typically ≥ 8.5 years for standard sequential approach).

**Conclusions:**

In this study, we demonstrate that adaptive trial designs are suitable for TB regimen development, and we provide plausible design parameters for a platform adaptive trial. Ultimately trial design and specification of design parameters will depend on clinical trial objectives. To support decision-making for clinical trial designs in contemporary TB regimen development, we provide a flexible clinical trial simulation tool that can be used to explore and optimize design features and parameters.

**Supplementary Information:**

The online version contains supplementary material available at 10.1186/s12879-022-07846-w.

## Background

Tuberculosis kills more people than any other single pathogen. 1.2 million people died from TB in 2020 and, while this number was slowly decreasing in recent years from 1.7 million in 2000, progress was halted in 2020 with the first increase in TB mortality in decades as a result of the COVID-19 pandemic [1]. The unprecedented number of new drugs in development for the treatment of TB (more than fifteen in phase I or II clinical trials [https://www.newtbdrugs.org/pipeline/clinical, January 2022]) and the recent success of the 4-month regimen with rifapentine and moxifloxacin [2] provide hope that the 50-year-old 6-month first-line regimen for the world’s oldest disease could be replaced with shorter, safer, more effective regimens. Unlike previous approvals of single drugs for the treatment of TB, with limited information about use in combination [3], the development and approval of TB drugs is now focused on the combination regimen as a whole. A recent example would be the approval of a novel pretomanid-based regimen for the treatment of multidrug-resistant TB and extensively drug-resistant TB by the US FDA in 2019 and the EMA in 2020. Nonetheless, there are challenges in interpreting the data from the small uncontrolled trial that led to approval of the pretomanid-based regimen BPaL [4, 5], and consequent need for better trial designs.

With a rich pipeline of new drugs and urgent need for tools to end the TB epidemic, the conventional clinical development strategy of testing single substitutions in series is recognized as too inflexible, slow and resource intensive. With this strategy, it is impossible to evaluate all the new therapeutics and their combinations, increasing the likelihood of missing promising ones [6, 7]. Conversely, the efficiencies in adaptive clinical trial designs are well known in other disease areas [8], and the potential has been recognized in TB [9, 10]. A small number of TB clinical trials with adaptive designs have been initiated, including Simon’s two-stage design [11], a Multi-Arm Multi-Stage (MAMS) design [12] (NCT03474198), a Bayesian response-adaptive (BAR) design [13] (NCT02754756), and an adaptive dose-finding design (NCT04044001). Adaptive trial designs are frequently used in dose-finding trials but are also particularly effective in platform trials, where multiple interventions are simultaneously compared to a single control group. Each of these designs have advantages, limitations, and contexts of use that have not been comprehensively described and evaluated in the setting of TB drug development. Additionally, with funding levels for TB research and development at half of what is required [14], it is imperative that these trials employ designs that can quickly and accurately identify the most promising regimens to fund and continue development. These trials must also generate strong evidence to support regulatory approval, inclusion in international practice guidelines, and programmatic implementation.

Our objective was to conduct a clinical trial simulation study to evaluate and compare innovative late-stage clinical trial development pathways in TB drug development. We compared Phase II/Phase III sequential and seamless approaches, including Bayesian adaptive response and multi-arm multi-stage designs in terms of their efficiency and ability to identify successful regimens. We then provide recommendations for when, where, and how these innovative development strategies could be applied.

## Methods

### General considerations

We focused on the late-stage clinical development pathway from phase II to phase III. For any regimen entering this pathway, we assumed that all drugs had been shown to have promising anti-TB activity in early-phase clinical trial(s) and that adequate early clinical and non-clinical safety studies had been undertaken to permit evaluation of the combination regimens being given for up to 4 months. We assumed that all pathways had these five main characteristics.

1. The main objective of all pathways is to identify short treatment durations of 12 weeks or less that are noninferior to the control.
2. All pathways included the same control arm, the standard 6-month rifampicin-based regimen to benchmark the trial to historical data (necessary as culture conversion and other treatment response biomarkers vary even between studies of the same regimen [15]).
3. We did not consider more complex platform trial designs where treatment arms are added during the trial.
4. Recruitment to treatment arms can be stopped early for lack of benefit based on interim analysis results (design-dependent), but not for intermediate indicators of overwhelming efficacy. Potentially promising TB regimens based on interim analyses still require full enrollment of all patients to allow for precise estimates of efficacy.
5. All recruited patients in phase II are followed up to 78 weeks post-randomization to collect data on phase III clinical endpoints, e.g. TB-related unfavorable outcomes. Application of this standard feature of phase III designs allows for the data-enrichment of phase II designs and enables better learning opportunities [16].

### Clinical trial designs

**Fig. 1:**
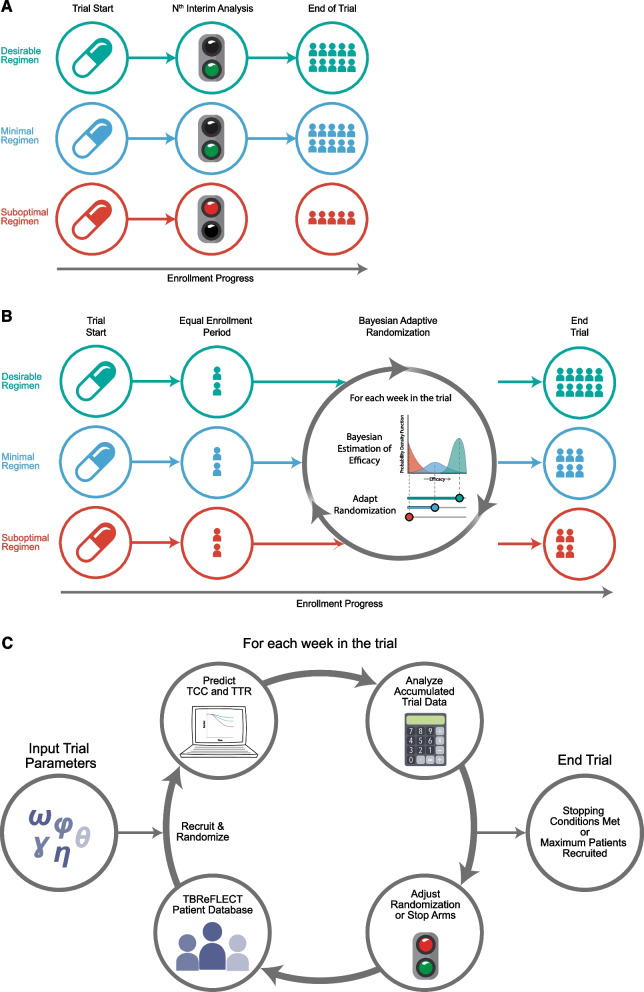
MAMS, BAR, & Simulation workflow schematics. **A** Basic schematic of Multi-Arm Multi-Stage adaptive trial design, where each experimental regimen must pass a predefined criteria at interim analysis to continue recruitment. **B** Basic schematic of Bayesian Adaptive Randomization adaptive trial design, where each week the efficacy of each experimental arm is estimated and randomization probabilities are weighted in favor of well performing arms. **C** Trial simulation workflow schematic, where simulation trial and design parameters were inputted and for each week in the trial patients are recruited and randomized, their individual TCC and TTR are calculated, and accumulated trial data is analyzed, and then randomization is adjusted or arms are stopped as needed. This is repeated until trial stopping conditions are met or the maximum number of patients are recruited

We evaluated two main adaptive trial designs, the Multi-Arm Multi-Stage (MAMS) and Bayesian Response Adaptive Randomization (BAR) designs (described below). These designs are currently in use for TB regimen development (NCT03474198 and NCT03259269 [13]) which gives evidence that they are considered by TB clinical trialists as suitable innovative approaches. The setting of phase III is non-inferiority as compared to control. We have evaluated five distinct drug development pathways:

A. Conventional sequential TB regimen development. The standard clinical development pathway takes a single combination regimen candidate through a learning phase II using an intermediate endpoint, then a confirmatory phase III using the final clinical endpoint. The pretomanid-moxifloxacin-pyrazinamide (PaMZ) regimen was the first combination regimen to follow this pathway and will therefore be our non-adaptive comparator case study.
B. Multi-arm multi-stage (MAMS) phase IIC with a separate phase III. This design evaluates several potential regimens with the objective to quickly identify poorly performing arms and stop enrollment to them. A fixed number of interim analyses are conducted with each intervention arm compared to control using an intermediate endpoint (time to culture conversion) with recruitment terminated to arms with insufficient evidence of benefit according to prespecified criteria (Fig. 1A). The MAMS trial design adapted for the context of TB has been previously described [10, 12].
C. Bayesian response adaptive randomization (BAR) phase IIC with a separate phase III. The objective is to continuously evaluate the efficacy of regimens and enroll more patients and gather more data about the most promising regimens. The BAR trial design adapted for the context of TB has been previously described [13, 17], where accumulating intermediate endpoint data throughout the trial is evaluated each week of the trial with randomization probability weighted in favor of better performing arms (Fig. 1B).
D. Seamless MAMS phase IIC/III. Where the phase IIC designs will only enroll a maximum of 100 patients per arm, the seamless trial combines the learning phase II and the confirmatory phase III, relying on adaptive elements to stop poor experimental regimens while enrollment continues for promising experimental regimens until phase III sample size (400 + per arm) is reached. Compared to the phase IIC MAMS design, the larger sample size of the seamless MAMS trial permits more interim analyses and adequately powered evaluation of final clinical endpoint of each regimen. The longer trial duration also permits the accumulation of final clinical endpoint data to perform interim analyses of final clinical endpoint.
E. Seamless BAR phase IIC/phase III. The seamless BAR design utilizes the same framework as the phase IIc design, but with a larger maximum sample size and adaptive randomization modified to also depend on a later endpoint to simultaneously evaluate regimens by intermediate endpoint and final clinical endpoint.

Our goal was to describe the operating characteristics of the pathways that graduated desirable regimens (defined in the next paragraph) from phase II to phase III in at least 95% of simulations and suboptimal regimens in no more than 10% of simulations. Given the scarcity of regimens with excellent safety profiles with a high chance of substantial treatment shortening, we decided to limit the risk of falsely stopping a desirable regimen at the expense of graduating a suboptimal regimen to phase III in 10% of occasions. This is analogous to limiting the false positive error (Type I error) rate to 10% while maintaining a high power in phase II.

For seamless phase III trials, to control type I error to < 5%, designs were optimized so that < 5% of simulations graduate suboptimal regimens and demonstrate noninferiority. We used the 16-week suboptimal regimen (Arm 9) to evaluate type I error and a 5.5% noninferiority margin (the true difference between the median cure rate of Arm 9 and control, see Table 1) was selected for a two-tailed 95% confidence interval noninferiority test.

**Table 1:** Simulated regimens designed according to 2016 treatment shortening target regimen profile [18]

Arm	Regimen	Duration (Weeks)	Assumed TCC hazard ratio	Assumed TTR hazard ratio	Median cure rate (%)
0	Control (HRZE)	24	Reference	Reference	92.0
1	Desirable (meets targets)	8	3.4	0.6	90.0
2		12			92.5
3		16			93.5
4	Minimal (minimum targets)	8	1.9	0.7	86.5
5		12			87.5
6		16			90.5
7	Suboptimal (below minimum targets)	8	1.2	0.85	81.0
8		12			84.0
9		16			86.5

### Regimen characteristics

We assumed that the trial is evaluating 3 experimental regimens in comparison to the standard of care control arm, with the goal of treatment shortening from 24 to 16 weeks. The simulated regimens were designed to have various characteristics (desirable, minimal, suboptimal) with respect to WHO treatment target regimen profile shortening goals [18] (Table 1). The desirable and minimal regimens have efficacy comparable to the 24-week standard of care when given for 12 weeks and 16 weeks respectively. A suboptimal regimen has efficacy that is only slightly better than the 24 week standard of care when given for 24 weeks. Each experimental regimen is evaluated at 3 durations (8, 12, 16 weeks), which brings the total number of arms to 10. See Additional file 1: Fig. S1 for a graphical representation of the relationship between regimen potency, treatment duration, time to culture conversion, and time to TB-related unfavorable outcome. The relationship between time to culture conversion and time to TB-related unfavorable outcome is based on the relationship observed in TB-ReFLECT (Pooled databse of OFLUTUB, ReMOX, and RIFAQUIN fluoroquinolone trials) and is described further in the Data Generating Mechanism sections later in the methods and Additional file 5.

Median cure rate is drawn from 1000 simulations of 2000 patients per arm with equal representation of easy, moderate, and hard-to-treat subpopulations within and between each arm drawn from the TB-ReFLECT database. The top regimens in order from best to worst: Arm 3, 2, 6, 1

### Clinical endpoints

Time to culture conversion (TCC) of sputum liquid culture was used as the intermediate clinical endpoint and time to TB-related unfavorable outcome as the final clinical endpoint. TB-related unfavorable outcomes include treatment failure, relapse, and death for up to 78 weeks post-randomization; henceforth abbreviated as relapse or time to relapse (TTR) [19, 20] since relapses make up most of TB-related unfavorable outcomes. The BAR design utilizes Bayesian estimates of binary endpoints as a measure of efficacy, so individual patient TCC and TTR were converted into binary outcomes at week 8, 24, 52, and 78 labeled as treatment success at 8 weeks or TS-8, TS-24, TS-52, and TS-78.

### Data generating mechanism

The clinical trial data was simulated in R utilizing integrated parametric survival models [19] for intermediate endpoint TCC (up to 26 weeks) and final clinical endpoint TTR (up to 78 weeks). Sputum samples were taken from patients at 1, 2, 4, 6, 8, 12, 17, 22, 26, 39, 52, 65, and 78 weeks. The models quantify relationships between clinical, demographic and regimen features with phase II and phase III outcomes. To reliably represent the population of TB patients, we sampled patients with replacement from the TB-ReFLECT trial participant database with 3411 participants from the modified intent-to-treat analyses of three large TB phase III trials [19]. We used definitions of easy/moderate/hard-to-treat patient populations from Imperial et al. [20]. to weight the sampling so that all three risk strata groups are equally represented in each simulated trial.

The joint parametric survival models for intermediate (TCC) and final (TTR) endpoint were used to generate individual patient outcomes for each of the regimens. In the models, in addition to treatment effects, time to culture conversion was delayed by older age, higher smear grade, or African clinical site (vs. non-African). Patients with delayed time to culture conversion, male sex, HIV-positive status, or cavitation on baseline chest x-ray had higher risk for TB-related unfavorable outcomes (final endpoint). Patients with the same characteristics will have identical probability functions for TCC and TTR, however the actual observed times for each patient are not deterministic, but randomly and independently drawn from these probability distributions. Models are described in more detail in the supplement and in Imperial et al. [20]

### MAMS design parameters

There are three MAMS design parameters to optimize: number of interim analyses, interim timing, and interim criteria. The timing of the interim analysis is crucial; too early and too little data is available to make adequately confident decisions, too late and too many patients have been enrolled into poorly performing regimens. Interim criteria were framed as a minimum TCC hazard ratio and maximum absolute relapse rate (at 52 weeks) that each arm must not pass to continue enrolling until the end of trial. The control arm matches enrollment to the investigational arms and the trial continues until the maximum sample size is reached in arms that are not stopped. These parameters were explored in a grid-like fashion with the goal of maximizing the probability of stopping suboptimal regimens and minimizing the probability of stopping desirable regimens, with the earliest interim timing and the least number of interim analyses.

### Bayesian response adaptive randomization

The BAR design estimates efficacy in each arm after a week of participants are randomized and randomization probabilities are adjusted proportionally to the efficacy of each arm. Randomization into the control arm matches the experimental arm with the highest randomization probability, so that the two will have approximately equal sample sizes. Two stopping rules were used: (1) recruitment to an arm is stopped after reaching a maximum sample size (recruitment continues to the other arms and control), and (2) the trial ends when *n* arms reach maximum sample size, where we allowed n to range from 1 to 5. These rules were designed to recruit rapidly to the best performing arms and stop the trial as soon as the top *n* arms have recruited enough patients for sufficient statistical power to demonstrate efficacy.

We modified a Bayesian adaptive randomization framework that has been described elsewhere [13, 17], our modifications are briefly described here but also in more detail in the supplement. Two BAR tuning parameters, *ɣ* and *η*, determine how aggressively (*ɣ*) the BAR algorithm and when (*η*) adaptations to randomization probability are weighed in favor of well performing arms. We measured the aggressiveness of adaptive randomization by measuring the ratio of patients allocated to the 16-week desirable regimen over the 8-week suboptimal regimen, while the variability in patient allocation across arms between simulations was quantified by the % coefficient of variation (%CV) of that ratio. These two parameters were explored in a grid-like fashion across a range of reasonable values. Further, parameters were optimized to maximize aggressiveness of randomization and to minimize variability in allocation ratio between simulations with the goal of graduating clinically noninferior arms and stopping suboptimal arms. For phase IIc and seamless designs respectively, graduation of an arm was defined as greater than 80 and 350 patients recruited into the arm and stopping of an arm defined as less than 50 and 200 patients recruited (analogous to stopping at MAMS first and second interims respectively). The equal recruitment period (before the allocation ratio is changed by the algorithm) was adjusted in the range 10–50 patients per arm in the seamless design and fixed to 10 patient per arm in the phase IIc design due to its short recruitment period. The complete design parameter space explored for trial design optimization is summarized in Table 2.

**Table 2:** Simulation conditions, assumptions, and trial design parameter space

	MAMS		BAR	
Parameters	Phase IIc	Seamless II/III	Phase IIc	Seamless II/III
Maximum patients	100 per arm	400 per arm	100 per arm	400 per arm
Recruitment rate	10 patients/week		10 patients/week	
Culture and data lag time	6 weeks		6 weeks	
Proportion of easy/moderate/hard to treat sub-populations	0.33 | 0.33 | 0.33		0.33 | 0.33 | 0.33	
Intermediate and surrogate endpoints evaluated	TCC HR	TCC HR TS-52	TS-8 TS-24	TS-8 TS-24 TS-52
Number of interim analyses	1–2*	2–3*	–	–
Timing of interim analysis	10–100 patients*	100–400 patients*	–	–
Interim criteria	TCC HR threshold: 1.1–2.3* Relapse % threshold: 4–20%*		–	–
Equal recruitment period (before adaptive algorithm is initiated)	–	–	10 patients per arm	10–50 patients per arm*
Bayesian adaptive randomization tuning parameters	–	–	Nonaggressive–Aggressive ɣ = 1–25* η = 0.1–2.0*	
Trial stopping rules	–	–	4 Arms reach max N	3 Arms reach max N
Priors	–	–	–	Optimistic* Skeptical*

*Indicates trial design parameters that were explored, and optimized

### Simulation tool

Recruitment to the simulated trials was fixed at a total of 10 patients per week, selected as a reasonable estimate for a global multi-center TB trial [18–20]. It was assumed that TCC and TTR were available for statistical analysis 6 weeks after the actual event to account for the biological assay time and for the results to be entered into the database. For each week of the trial simulations:

1. 10 patients are ‘recruited’ from TB-ReFLECT patient database
2. Patients are randomized into arms/regimens; their individual intermediate (TCC) and final (TTR) endpoints simulated by the aforementioned integrated parametric survival models. Prior to any adaptive modifications, patients in each simulated trial were randomly allocated with equal probability to either a control arm or one of nine intervention arms.
3. For the BAR design, available data are analyzed, and randomization probabilities are updated. For the MAMS design, available data are only analyzed when interim analyses are triggered, then randomization is updated accordingly.
4. Proceed to the next week.
5. Steps 1–4 are repeated for each week of the trial until maximum number of patients have been recruited or trial stopping rules have been met.

MAMS and BAR design parameter space were explored with 1000 simulations performed for each set of parameter values to identify optimal sets with desired characteristics. Further details of design parameters have been explained above and the parameter space explored defined in Table 2; the simulation workflow is shown in Fig. 1C.

### Performance measures

Each of the five optimized clinical development pathways were compared based on the following performance measures: total study and pathway duration, total recruitment, enrollment per arm, bias in estimation of treatment effects, number of observed relapse events per arm, and the probability of correctly selecting treatment shortening arms and stopping undesirable arms.

### Sensitivity analyses

Sensitivity analyses were performed to assess how recruitment rate, the assumed relationships in the parametric survival models, longer data lag times, and different compositions of desirable, minimal, and suboptimal regimens in the trials affect our conclusions. Further details can be found in supplemental methods.

## Results

Results are summarized for each distinct design first, followed with a comparison between designs. Since differences in treatment duration impact the final endpoint and not the intermediate endpoint, treatment duration does not impact phase IIc stopping criteria (based on the intermediate endpoint of TCC) and therefore different durations of the same regimen are combined in the presentation of the phase IIc results.

### Multi-arm multi-stage (MAMS) phase IIc

**Fig. 2:**
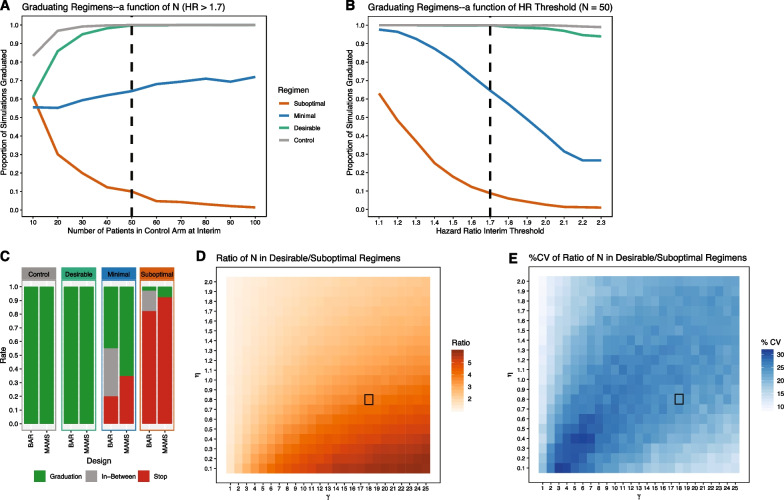
Phase IIc MAMS and BAR optimization and comparison. **A** Fraction of simulations graduated for Phase IIC MAMS trials with interim criteria fixed at TCC HR > 1.7 while changing interim timing from 10 to 100 patients recruited into the control arm, at 10 patients/week interim timing is approximately study week 10–100. **B** Fraction of simulations graduated for Phase IIC MAMS trials with interim timing fixed to 50 patients and changes TCC HR criteria from 1.1 to 2.3. Dotted lines show the chosen optimized conditions, where an interim timing of 50 patients per arm is the earliest timing in which the risk of stopping the desirable regimen is negligible and an interim criteria of TCC HR > 1.7 is the strictest criteria in which the risk of stopping the desirable regimen is negligible. The control in grey, represents the proportion of simulations in which the trial was not stopped prematurely due to all investigational arms being stopped. **C** Comparison of graduation and stopping rates of optimized Phase IIc MAMS and BAR designs. The continuous nature of the BAR recruitment was translated into a semi-discrete outcome for comparison to the MAMS design, graduation of an arm was defined as greater than 80 patients recruited into the arm, stopping of an arm defined as less than 50 patients recruited (analogous to stopping at MAMS interim) and in-between defined as 50–80 patients recruited into the arm. Both designs meet our target criteria graduating > 95% of desirable regimens and < 10% of suboptimal regimens. **D** Heatmap quantifying the aggressiveness of Bayesian adaptive randomization as the ratio of patients allocated to the desirable regimen/suboptimal regimen across a range of reasonable ɣ and η values. **E** Heatmap of the variability expressed as %CV in the ratio shown in D across 1000 simulations. The optimal condition outlined in black was chosen for its aggressiveness and limited variability while meeting our target critiera

We found that one interim analysis that occurs after 50 patients (Fig. 2A) have been recruited in each arm and an interim criteria hazard ratio threshold of 1.7 (Fig. 2B) meets our criteria for an optimal design. In this setting, the desirable and suboptimal regimens graduated in 99.8% and 8.2% of simulations respectively. Minimal regimens graduated in 65.2% of simulations (Fig. 2C). Setting a lower hazard threshold or an earlier interim analysis will decrease the graduation rate of desirable regimens without appreciably increasing the stopping rate of suboptimal regimens. A second interim was explored but did not add value. With the suboptimal regimen already being stopped in 91.8% of simulations after the first interim analysis, the logistical cost of a second interim analysis did not justify the small potential benefit of stopping additional suboptimal regimens.

### Bayesian response adaptive randomization (BAR) phase IIc

We identified a set of BAR tuning parameter values (*ɣ* = 18, *η* = 0.8) that meets our criteria for an optimal design. This optimized BAR design aggressively randomizes 2.5 times (Fig. 2D) more patients into the desirable regimen (median N = 100, see Additional file 1: Fig. S2B) over the suboptimal regimen (median N = 40) while limiting % CV of this ratio to less than 20% (Fig. 2E). The desirable regimen graduated in 99.5% of simulations (where graduation is defined as the sample size reaching 80 or more), and was stopped in 0.1% of simulations (where stopping was defined as the sample size not exceeding 50, Fig. 2C). For minimal regimens 49.5% graduated and 18.3% were stopped. For suboptimal regimens 2.7% graduated and 81.0% stopped. The heatmap in Fig. 2D shows the *ɣ* and *η* parameter space explored to optimize the aggressiveness of adaptive randomization, other sets of *ɣ* and *η* values might be suitable for trials with different objectives.

### Seamless MAMS phase II/III

**Fig. 3:**
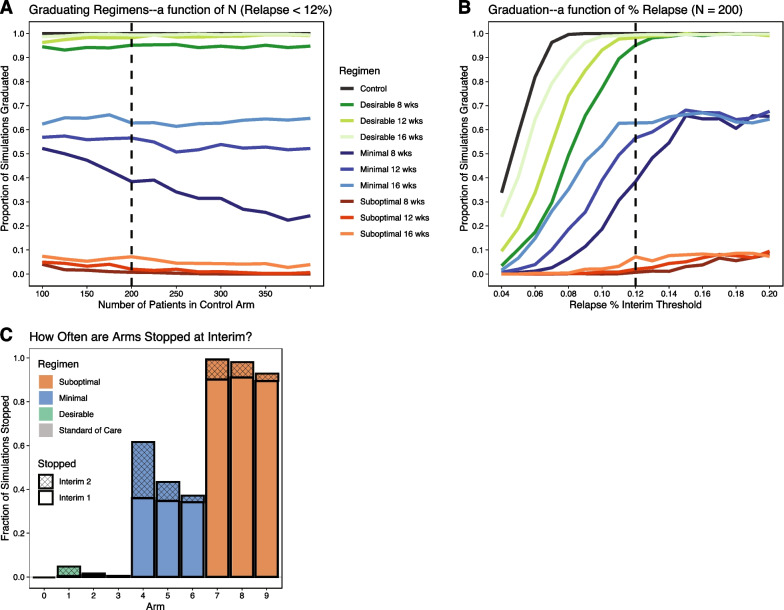
Seamless MAMS interim timing and criteria optimization **A** Fraction of simulations graduated for Seamless MAMS trials with second interim criteria fixed at relapse rate < 12% while exploring second interim timing. The control in grey, represents the proportion of simulations in which the trial was not stopped prematurely due to all investigational arms being stopped. **B** Fraction of simulations graduated for Seamless MAMS trials with second interim timing fixed at 200 patients per arm while exploring second interim criteria. Dotted line represents selected optimized second interim conditions, where an interim timing of 200 patients per arm is the earliest timing in which the risk of stopping the desirable regimen is negligible and an interim criteria of relapse rate < 12% is the strictest criteria in which the risk of stopping the desirable regimen is negligible. **C** Fraction of arms stopped in interim 1 and 2. Note that interim 2 can distinguish between different durations of the same regimen, whereas interim 1 cannot

Building off the optimized phase IIc MAMS design, we found that a second interim occurring after 200 patients (Fig. 3A) have been enrolled in each continuing arm with an interim criteria of a 12% relapse threshold (Fig. 3B) met our criteria for an optimal seamless design. Because the differences in treatment duration largely manifest as differences in relapse rate, a relapse rate threshold is able to distinguish between different durations of the same regimen. Therefore, the second interim served primarily to stop regimens that have a favorable intermediate endpoint (TCC) profile, but underwhelming efficacy measured by final endpoint, e.g. relapse rate (Fig. 3C). Under these conditions, desirable regimens at 8, 12, and 16 week durations have a 2.2%, 1.3%, and 0% chance of being stopped respectively, minimal regimens 65.8%, 38.6%, and 37.5%, and suboptimal regimens 99.7%, 97.5%, and 95.3%. Only 9/1000 (0.9%) simulations graduated and demonstrated noninferiority for the 16-week suboptimal regimen (analogous to type I error). A third interim was explored, but since a two interim design well exceeded our aforementioned trial objective of graduating > 95% of desirable regimens and < 10% of suboptimal regimens, a third interim would provide limited benefit and was considered unnecessary.

### Seamless BAR phase II/III

**Fig. 4:**
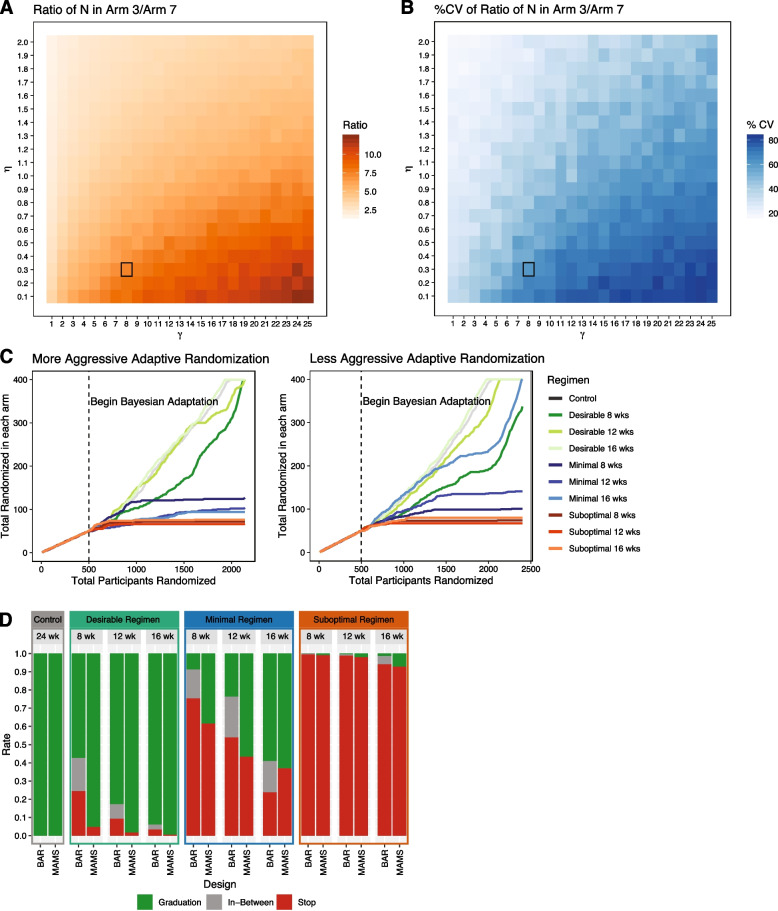
Seamless BAR optimization. **A** Heatmap quantifying the aggressiveness of Bayesian adaptive randomization across a range of ɣ and η values. **B** Variability of patient distribution across simulations represented as % CV. **C** Representations of aggressive and less-aggressive BAR designs shows the suitability of less-aggressive adaptation for the seamless design. Less-aggressive adaptation allows relapse data to be taken into account and adjust randomization. At 1100–1200 patients randomized Arm 1 recruitment slows down while Arm 6 speeds up, reflecting incoming relapse data that reduces confidence in Arm 1 and increases confidence in Arm 6. **D** Graduation and stop plot comparing Seamless MAMS and BAR. The continuous nature of the BAR recruitment was translated into a semi-discrete outcome for comparison to the MAMS design, graduation of an arm was defined as greater than 350 patients recruited into the arm, stopping of an arm defined as less than 200 patients recruited (analogous to stopping at MAMS interim) and in-between defined as 200–350 patients recruited into the arm. Both designs are comparable and are suitable for our purposes, but BAR excels at penalizing and thus stopping poorly performing arms

We found that less aggressive BAR tuning parameters (*ɣ* = 8, *η* = 0.3) compared to the more aggressive phase IIc BAR tuning parameters, were better suited for our criteria for an optimal seamless trial. This optimized BAR design randomizes 5.9 times (Fig. 4A) more patients into the 16-week desirable regimen (median N = 400) over the 8-week suboptimal regimen (median N = 67) while limiting the variability across simulations to < 50% (Fig. 4B). Although the heatmap (Fig. 4A) reveals more aggressive options for *ɣ* and *η*, the goals of phase IIc and seamless phase II/III differ where a seamless trial must not only distinguish between different durations of the same regimen but also evaluate regimens by the 52-week relapse endpoint. We found that a moderately aggressive randomization was more suitable for this purpose; in Fig. 4C, representations of aggressive and less-aggressive BAR simulations demonstrates that less-aggressive adaptation allows more time for 52-week relapse data to accumulate and therefore affect randomization. At 1100–1200 patients randomized, the 8-week desirable regimen recruitment begins to slow down while the 16-week minimal regimen speeds up in the less aggressive design eventually overtaking 8-week desirable regimen recruitment as compared to the more aggressive design. This is the consequence of the accumulation of incoming long term relapse data and demonstrating the benefit of slower adaptation in this context. Additional file 2: Fig S3 further demonstrates the desired behavior where the enrollment distribution in each arm produced a graded allocation of patients across the different treatment durations where the phase IIc design did not.

The desirable regimens at 8, 12, and 16 week durations graduated in 57.2%, 82.6%, and 93.8% of simulations respectively and stopped in 24.5%, 9.4%, and 3.5% of simulations respectively (Fig. 4D). Although this set of design parameters falls just short of our 95% graduation target, the graded response of the BAR design makes a difficult translation to a binary graduation-stop result and we believe that this set of parameters is suitable for our trial objectives. Minimal regimens at 8, 12, and 16 week durations graduated in 8.6%, 23.5%, and 58.9% of simulations respectively and stopped in 75.5%, 54.1%, and 23.9% of simulations respectively. Suboptimal regimens graduated in 0%, 0% and 1.3% of simulations and stopped in 99.6%, 98.9%, and 94.2% of simulations. Additionally, only 3.5% of simulations falsely rejected the null hypothesis for the 16-week suboptimal regimen (analogous to type I error).

### Overall comparison of trial designs

**Fig. 5:**
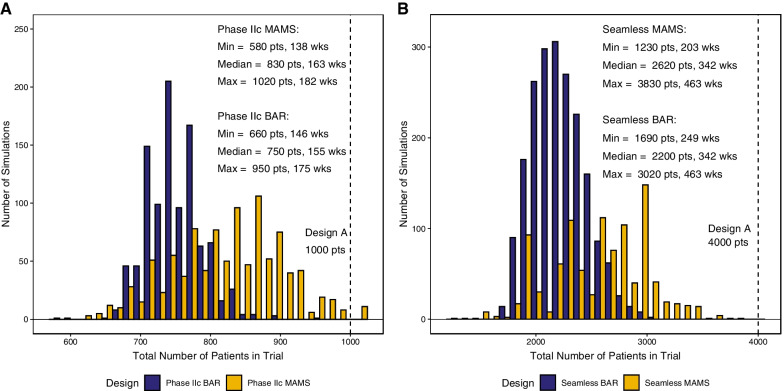
Study duration and total recruitment comparison of BAR and MAMS. **A** Phase IIc and **B** Seamless Phase II/III comparisons. BAR and MAMS designs have comparable performance in graduating the best regimens and stopping the suboptimal regimens (demonstrated previously) while BAR consistently outperforms MAMS in study duration and total recruitment. Although both MAMS and BAR provide enormous time and patient savings compared to conventional clinical trials, the median Phase IIc BAR enrolls 80 fewer patients and saves 8 weeks of time compared to the Phase IIc MAMS and the median Seamless BAR enrolls 420 fewer patients and saves 42 weeks of time compared to the Seamless MAMS

BAR designs offer a clear advantage in identifying the most promising regimens more quickly with fewer patients than MAMS designs; BAR designs recruited 80 and 420 patients less in phase IIc and seamless trials respectively. Both MAMS and BAR designs were much more efficient compared to the conventional sequential approach which would recruit 1000 patients for phase IIc and 4000 for the seamless design (Fig. 5). A major contributing factor to the observed recruitment advantage of the BAR design, is that for MAMS, patient recruitment by arm is clustered around the interim analyses (50 and 200 patients) and trial end (400 patients), while BAR’s patient distributions are continuous with each arm’s median scaling with efficacy (Additional file 1: Fig. S2B and Additional file 2: Fig. S3). Both MAMS and BAR designs produce accurate efficacy estimates consistent with values produced by unbiased simulations (Additional file 3: Fig. S4A and C), however the precision in estimating efficacy is directly proportional to the number of observed relapse events (Additional file 3: Fig. S4B) and thus number of patients (Additional file 2: Fig. S3) randomized into that arm. None of the designs introduced significant bias (< ± 7% median bias, Additional file 3: Fig. S4C) except for 12% median underestimation of relapse rate in Phase IIc BAR suboptimal regimens due to the small sample size (N = 40).

### Sensitivity analyses

Recruitment rates of 5–15 patients per week did not significantly change the results of the optimized adaptive trial designs, MAMS and BAR graduation rates changed less than an absolute 5%. However, a faster enrollment of 30 patients per week was too fast to allow for TCC or relapse data to accumulate in time for MAMS interim analyses or significantly affect BAR randomization probabilities. BAR designs were more sensitive to changes in recruitment rate because graduation is dependent on patient randomization which is directly dependent on the accumulation of data. All graduation decisions benefitted from a slower recruitment rate, but the benefit was minimal in the range 5–15 patients per week. Sensitivity analyses are presented in more detail in Additional file 5.

## Discussion

In this study, we have demonstrated the suitability of adaptive trial designs for TB regimen development and have described optimal MAMS and BAR designs in the phase IIC and seamless phase II/III settings. In the seamless phase II/III designs, suboptimal regimens graduated in less than 10% of simulations with desirable regimens graduating in more than 95% of simulations. Both designs were able to discriminate between different durations of the same regimens where the 16-week minimal regimen graduated in 62% and 58.6% of simulations and the 8-week minimal regimen graduated in 33% and 8.6% of simulations for MAMS and BAR designs respectively. Additionally, both designs were able to reliably select the noninferior regimens (12 and 16 week desirable regimen) in at least 80% of simulations; bias was minimal in arms that graduated. In the phase IIC designs, desirable regimens graduated in > 99% of simulations, while suboptimal regimens graduated in 8% and 2.7% of simulations for the MAMS and BAR designs respectively. Importantly, since adaptation in the smaller phase IIC trials is based only on the intermediate endpoint, TCC, which is independent of treatment duration, these phase IIc designs were not able to discriminate between different durations of the same regimen.

### Recommendations regarding BAR and MAMS

Our objective was to compare the operating characteristics of different designs that have utility for TB regimen development, and specifically to describe particular designs that met our objectives of a typical regimen development program. From a broad view, adaptive trial designs offer a clear advantage in simultaneously evaluating more intervention arms with similar numbers of patients in a shorter time frame. Indeed, we found that the BAR designs require the least number of patients and also allow for more flexibility in trial objectives. For example, the ɣ and η parameters of the BAR design can be modified to have a less aggressive adaptive algorithm so that less weight is put on early data and the algorithm waits for more data before substantially changing randomization probabilities. Importantly, the adaptation algorithm in BAR weighs randomization in favor of the best performing arms relative to control. Therefore, while there is not necessarily an direct comparison with the other arms, the final sample sizes and trial durations are influenced by the indirect comparative efficacy of the arms. In other words, a trial with all equivalent suboptimal regimens will randomize patients equally between arms, as will a trial with all equivalent desirable regimens. Therefore, the BAR design is most efficient for multi-arm trials with a combination of regimens of unknown efficacy potentially spanning suboptimal to desirable. In contrast, the MAMS design depends only on comparisons against the control arm and therefore whether a particular arm stops or continues does not depend on other arms in the trial, although that doesn’t rule out comparisons between two active arms in an indirect fashion. Additionally, MAMS designs are more efficient and result in smaller sample sizes compared to BAR when all evaluated treatments are underperforming and fail to achieve minimum efficacy targets [21, 22].

Critically, the choice of design and specification of design parameters, including timing of the interim analysis and target efficacy thresholds for the MAMS design, and randomization tuning parameters and stopping rules for the BAR design, will very much depend on the sponsor’s objectives. If the objective is to consider each regimen on its own merit as compared to control, then the MAMS design is better suited, but if the objective is to rapidly select amongst a number of regimens that are likely to include a range of regimens from suboptimal to desirable then the BAR design is perhaps better suited. In the current landscape of TB drug development, with over ten new drug candidates, a MAMS design would select all regimens that meet the minimum target criteria, while the BAR design would efficiently rank the regimens and recruit greater numbers of patients to the top regimens to generate further evidence of efficacy. If the BAR design is modified to permit stopping for futility (see section on additional design modifications below) then the benefits (and limitations) of both MAMS and BAR would likely be combined—exploration of this was beyond the scope of this paper.

There are other considerations of MAMS and BAR designs that cannot be described with clinical trial simulations and make each design more or less suited to trial objectives. Adaptations can only occur at a limited number of interim analyses in the MAMS design which means a data and safety monitoring board can still have oversight over stopping decisions and incorporate safety considerations in their deliberations. Ongoing adaptation in the BAR design adds additional burdens on clinical trial conduct, workflow, and data management to ensure that data is available on the database with as few errors as possible at each point throughout the trial [23], whereas this is only needed at specific times when interim analyses are being conducted in the MAMS design. On the other hand, MAMS can be less efficient than a BAR design when there are large differences between regimens as no adaptations can occur until the first interim analysis.

The typical delay in culture assay results (up to 6 weeks to perform assays) introduces some complexities in implementing adaptive trial designs in TB clinical development. Ideally, adaptive trial designs base interim analyses on early, accurate, and fast prognostic biomarkers. TCC is anything but early and fast, with most patients’ culture converting between 4 and 8 weeks and therefore assay results not available until 10–14 weeks post-treatment initiation. Nevertheless, the follow up period is long, up to 78 weeks, which in combination with the slow recruitment rate (approximately 10 per week for a large global multicenter trial) allows for plenty of time for the adaptive elements of a trial design to be effective even with a slow intermediate endpoint like TCC.

Notably, seamless designs are especially time efficient; in addition to the time saved from reduced patient enrollment and thus reduced enrollment period, moving seamlessly between phases eliminates the need for two treatment follow up periods (each 18 months after enrollment of the last patient) and a 12–18 month analysis and planning period between trial phases [24]. However, seamless designs require careful planning, rapid and efficient data management, large upfront investment of logistics and resources, and steadfast sponsors and stakeholders to complete a prescribed seamless trial, not all of which are usually present which means seamless designs are rare in practice. In total, the seamless approach can accelerate TB regimen clinical development timelines by 2–4 years.

### The effect of recruitment rate

We have shown that modest changes in recruitment rate have limited impact in the scenarios we explored. It is true that a substantial increase in recruitment rate can reduce efficiencies in adaptive designs with late outcome measurements, but this effect is limited unless recruitment is very fast relative to the overall study size. This means that it would normally be worth adding a new site to increase the rate of recruitment while reducing the overall duration of the trial with only a modest impact on design efficiencies.

### Additional design modifications

Besides the parameters and designs described thus far, there are many additional levers and interesting tools that can be adapted to suit various objectives: (1) Adding new regimens and arms in an ongoing phase IIc screening trial is of particular interest and there are case studies in oncology and COVID where this has been done with a BAR [25] or MAMS [26, 27] design. (2) Stopping for overwhelming efficacy/futility is another often considered rule. Although stopping for overwhelming efficacy was not considered within the context of our work due to potential power issues, this feature can easily be added to MAMS interim analyses (which stop for futility). Stopping for overwhelming efficacy/futility can also be added to a BAR design, instead of only relying on the adaptive randomization algorithm, thereby attaining some of the benefits of the MAMS design. However, these features must be implemented with planned interim analyses and appropriate sample size adjustments to control type I error [28–30]. (3) REMoxTB, RIFAQUIN, OFLUTUB, and ACTG5349/Study 31 phase III trials have all demonstrated the complex interplay between regimen potency, disease severity, and treatment duration [19]. Patient subpopulation enrichment which enrolls and enriches for hard or easy-to-treat patients—is a strategy employed to control the disease severity representation in the trial. It can be used to increase trial efficiency and likelihood of success. Hard-to-treat subpopulations have a higher probability for treatment failure and bacteriological relapse thus, in this population, one might observe the same number of unfavorable events with a smaller sample size. Opting to enrich for hard-to-treat subpopulations instead of including all patients must be carefully approached. While higher outcome rates are likely to be observed, and therefore the design will have greater power to distinguish regimens, these types of trials might have other limitations when extrapolating efficacy and safety findings to the unstudied patient subpopulations. (4) Finally, treatment duration represents another manipulable variable which heavily impacts regimen efficacy and trial success, and TB trialists have turned to duration randomization trials [31, 32] to optimize treatment duration prior to large confirmatory trials. The trial designs described here can be altered to estimate duration response curves or to select the shortest treatment duration that exceeds a minimum likelihood threshold for noninferiority. Each of these design modifications could change operating characteristics of MAMS and BAR trials; additional simulations would be needed to explore this.

### Limitations

There are a few limitations in the work described here. First, we explored a limited set of possible arm and regimen configurations, along with a limited set of parameters and trial rules. Given the vast parameter space and the flexibility of trial designs, we wanted to limit the scope of this study within the context of a large platform adaptive trial while highlighting the possible mechanisms that can be adjusted for a different set of trial objectives. Second, the models used to predict individual patient intermediate and final endpoints are based on data generated from rifamycin containing regimens, and the risk factors for culture conversion and relapse may not hold true for novel regimens with a different mechanism of action. However, with the recent success of high dose rifapentine with moxifloxacin in Study 31/A5349, it is clear that rifamycin containing regimens will remain first line for drug sensitive TB for the foreseeable future. Finally, although we have confirmed that desirable regimens graduate in > 95% of simulations (analogous to power) and suboptimal regimens graduate and demonstrate noninferiority in < 5% of simulations (analogous to type I error), an in-depth power and type I error analysis was not explored in this study. To that end, we have assumed that a sample size of 400 is sufficient for a noninferiority confirmatory trial with a 6.6% margin to achieve 80% power and limit type I error to 5%. Instead, we focused on optimizing and reviewing the graduating and stopping decisions of the five pathways, which remain previously undiscussed in the context of TB.

## Conclusions

We have developed a flexible clinical trial simulation tool integrated with parametric survival models to accurately simulate the potential range of real-world trial outcomes [20]. We have also demonstrated the efficiencies of MAMS and BAR designs over conventional approaches for a platform adaptive trial and provided sets of optimized design parameters. Through our ongoing collaborations, this work described here will be used by international consortia for TB regimen development and sets the stage for future adaptive trial design studies within the context of TB.

## Supplementary Information

**Additional file 1: Figure S1.** Simulated Regimens Time to Relapse Kaplan Meier Estimates stratified by Time to Culture Conversion. In green are patients whose time to culture conversion is ≤ 4 weeks, in blue is > 4 and ≤ 8 weeks, in yellow is > 8 and ≤ 16 weeks, and in red is > 16 and ≤ 25 weeks. On the right are density plots of time to culture conversion for each of the regimens, more potent the regimens cause patients to culture convert earlier and thus the distribution becomes more right skewed. Together, the plots demonstrate the relationship between regimen potency, treatment duration, time to culture conversion, and time to relapse.

**Additional file 2: Figure S2.** Phase IIc Supplement. (A) points and the error bars represent the mean simulation HR estimation and 95% CI respectively. The accuracy of the HR estimate improves with increasing number of patients; interim timing at N = 50 is where the median estimate stabilizes and provides sufficient accuracy to make interim decisions. (B) Histogram of patient enrollment per simulation in each regimen across 1000 simulations of optimized BAR and MAMS trials. BAR’s graded response is clearly demonstrated in the median enrollment in each of the regimens. Within each regimen, BAR simulations also produce a distribution of patient enrollment, contrasting with MAMS simulations with patient enrollment clustered around 50 and 100 (interim and trial end).

**Additional file 3: Figure S3.** Seamless Phase II/III Supplement. (A) Histogram of patient enrollment per simulation in each arm across 1000 simulations of optimized BAR and MAMS trials. BAR’s graded response is clearly demonstrated in seamless designs as well.

**Additional file 4: Figure S4.** Comparison of Performance Measures. (A) Estimated relapse rate across arms and trial designs is accurate when compared to unbiased simulations. Lower accuracy and higher 95% prediction intervals are observed in arms with very low sample size, i.e. phase IIC BAR arms 7, 8, 9. (B) The higher number of relapse events observed in BAR designs’ desirable regimens provide greater evidence of efficacy for the best regimens. Given the low relapse rate in better regimens, higher sample size is particularly needed in the best regimens to observe an appreciable number of relapse events to produce an accurate relapse rate estimate. (C) Minimal bias is observed in estimation of relapse rate across designs, except for phase IIC BAR arms 7, 8, 9 where the low sample size produces a consistent underestimation of relapse rate. Overall, both MAMS and BAR designs provides excellent accuracy and precision in relapse rate estimation. The bias in suboptimal regimens is inconsequential as these arms would quickly be abandoned for lack of efficacy.

**Additional file 5.**

## Data Availability

The clinical trial simulation tool and datasets generated during and/or analyzed during the current study are available from the corresponding author on reasonable request. The standardized data for the OFLOTUB (ClinicalTrials.gov number NCT00216385), REMoxTB (ClinicalTrials.gov number NCT00864383), and RIFAQUIN (ISRCTN number 44153044) trials that support the findings of this study are publicly available to qualified researchers through the Platform for Aggregation of Clinical TB Studies (TB-PACTS).

## Abbreviations

TB: Tuberculosis
US: United States
FDA: Federal Drug Administration
BPaL: Bedaquiline, Pretomanid, Linezolid regimen
MAMS: Multi-arm multi-stage
BAR: Bayesian response adaptive randomization
PaMZ: Pretomanid, moxifloxacin, pyrazinamide regimen
TCC: Time to culture conversion
TTR: Time to relapse
%CV: Percent coefficient of variation
HR: Hazard ratio
TS-X: Treatment success at week X
