# Supplementary figures and images for: A comparison of clinical development pathways to advance tuberculosis regimen development
Source: BMC Infect Dis. 2022 Dec 9;22:920. doi: 10.1186/s12879-022-07846-w (PMC9733404; doi:10.1186/s12879-022-07846-w)

Proportion without TB-related outcomes

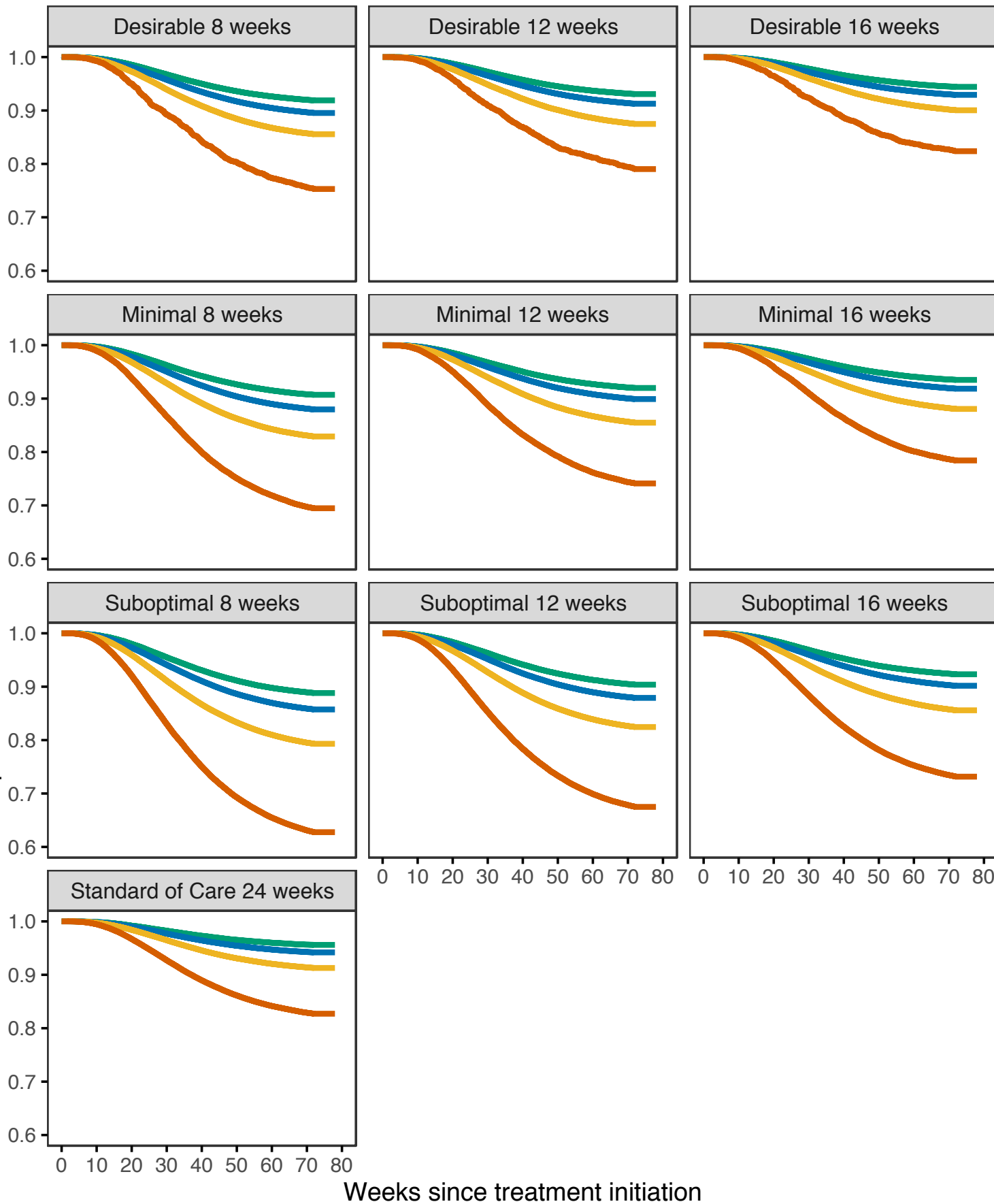

TCC 0-4 wks TCC 4-8 wks TCC 8-16 wks TCC 16-25 wks

Density

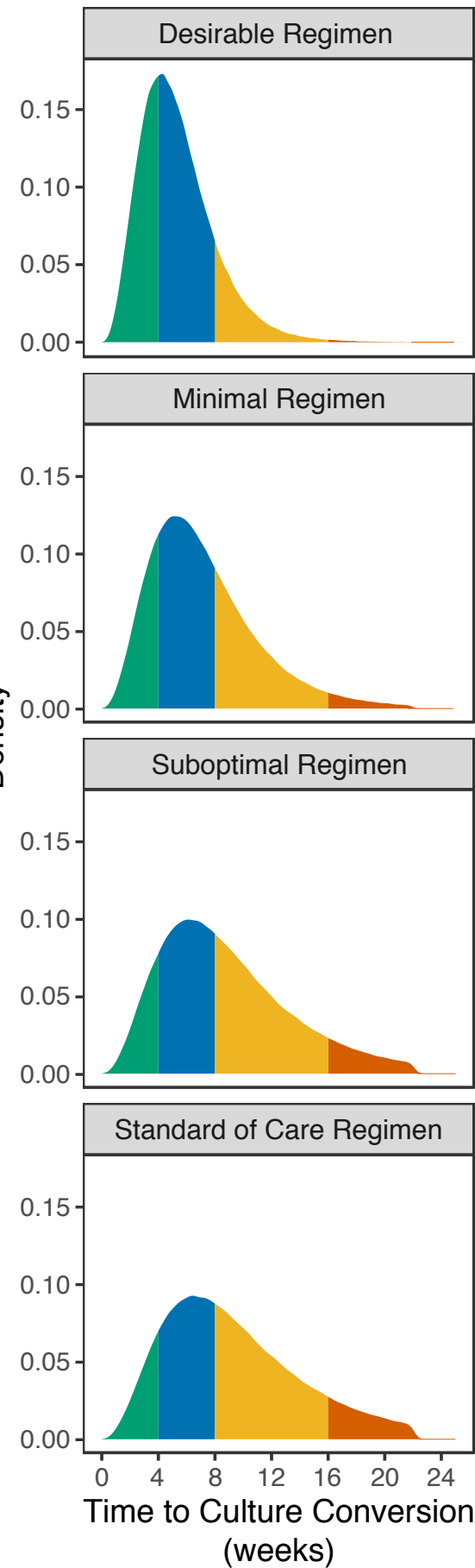

Supplement: Supplementary file 1 — Additional file 1: Figure S1. Simulated Regimens Time to Relapse Kaplan Meier Estimates stratified by Time to Culture Conversion. In green are patients whose time to culture conversion is ≤ 4 weeks, in blue is > 4 and ≤ 8 weeks, in yellow is > 8 and ≤ 16 weeks, and in red is > 16 and ≤ 25 weeks. On the right are density plots of time to culture conversion for each of the regimens, more potent the regimens cause patients to culture convert earlier and thus the distribution becomes more right skewed. Together, the plots demonstrate the relationship between regimen potency, treatment duration, time to culture conversion, and time to relapse. [file 12879_2022_7846_MOESM1_ESM.pdf]

**A**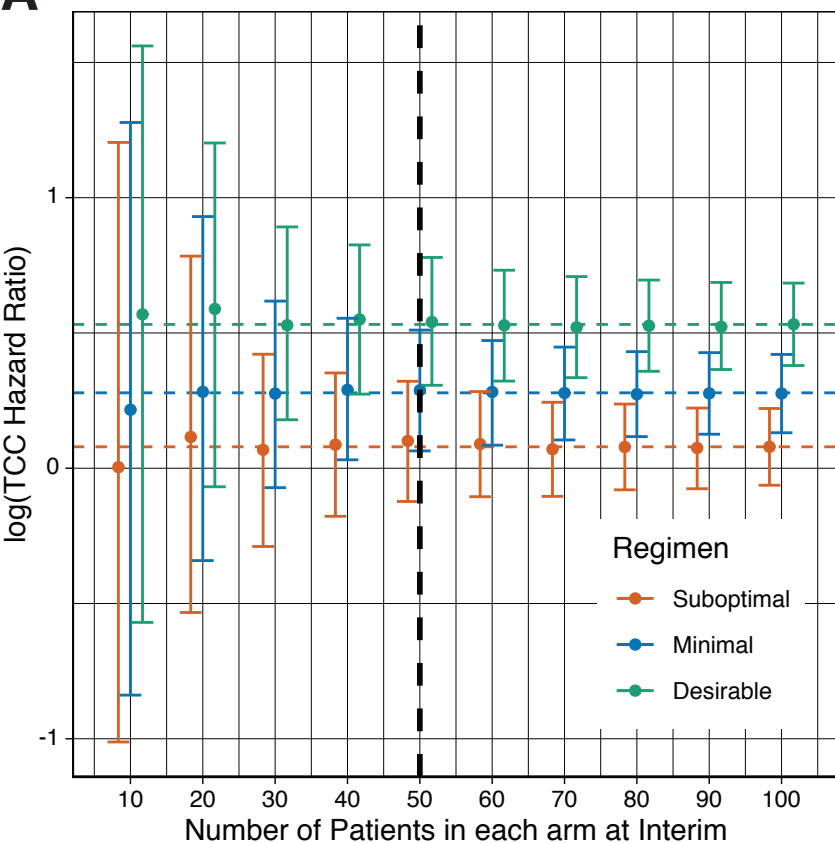**B**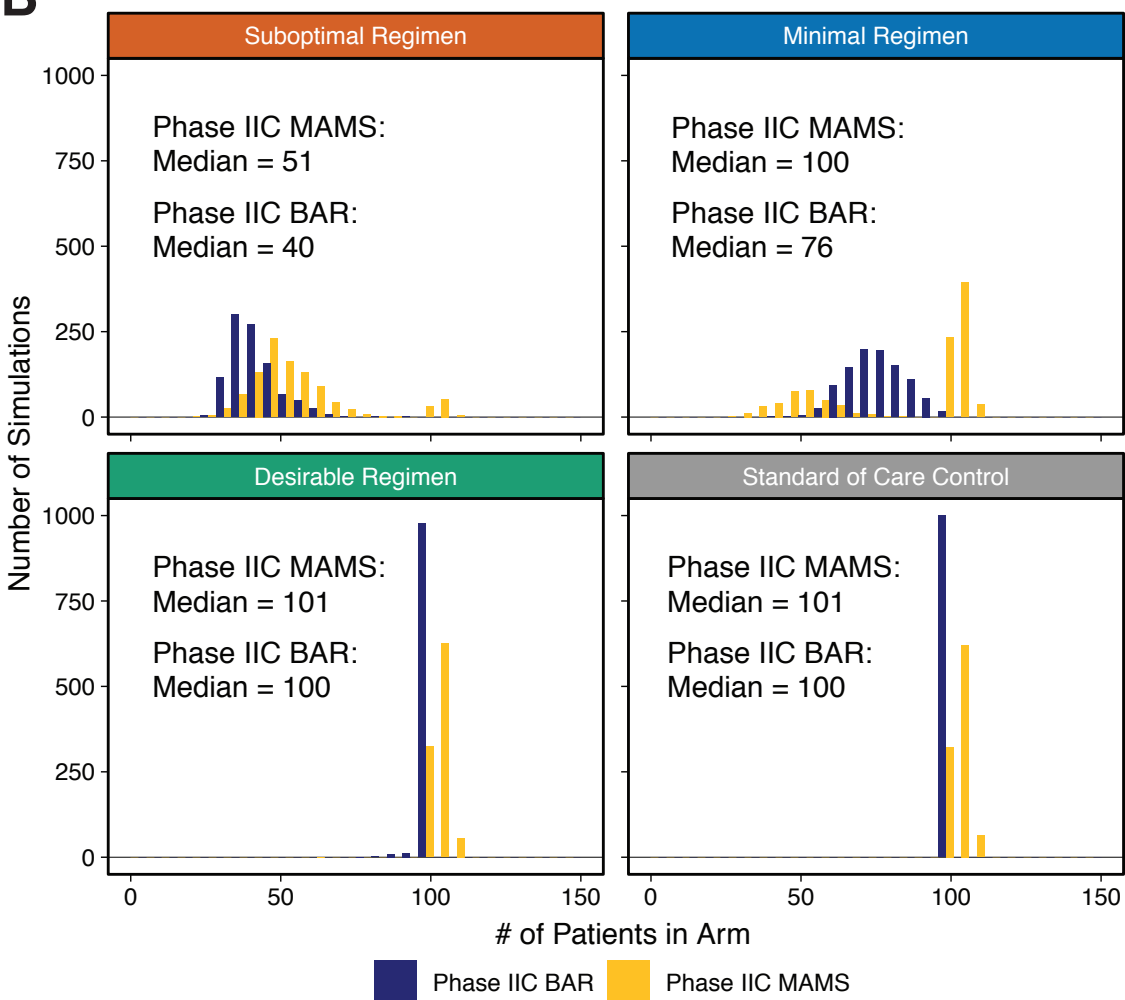

Supplement: Supplementary file 2 — Additional file 2: Figure S2. Phase IIc Supplement. (A) points and the error bars represent the mean simulation HR estimation and 95% CI respectively. The accuracy of the HR estimate improves with increasing number of patients; interim timing at N = 50 is where the median estimate stabilizes and provides sufficient accuracy to make interim decisions. (B) Histogram of patient enrollment per simulation in each regimen across 1000 simulations of optimized BAR and MAMS trials. BAR’s graded response is clearly demonstrated in the median enrollment in each of the regimens. Within each regimen, BAR simulations also produce a distribution of patient enrollment, contrasting with MAMS simulations with patient enrollment clustered around 50 and 100 (interim and trial end). [file 12879_2022_7846_MOESM2_ESM.pdf]

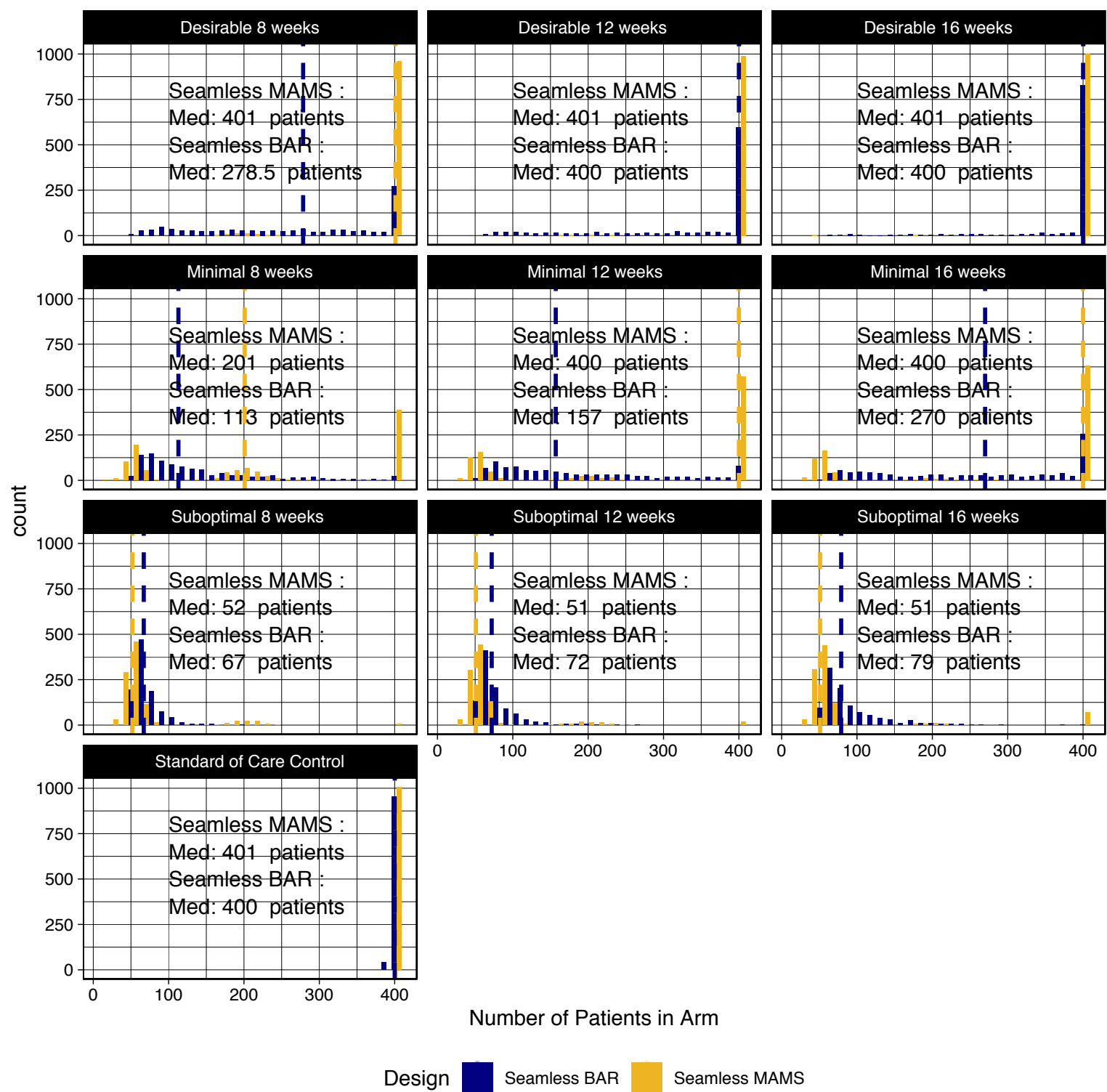

Supplement: Supplementary file 3 — Additional file 3: Figure S3. Seamless Phase II/III Supplement. (A) Histogram of patient enrollment per simulation in each arm across 1000 simulations of optimized BAR and MAMS trials. BAR’s graded response is clearly demonstrated in seamless designs as well. [file 12879_2022_7846_MOESM3_ESM.pdf]
