# Supplementary material for: A comparison of clinical development pathways to advance tuberculosis regimen development
Source: BMC Infect Dis. 2022 Dec 9;22:920. doi: 10.1186/s12879-022-07846-w (PMC9733404; doi:10.1186/s12879-022-07846-w)
Supplement: Supplementary file 5 — Additional file 5. [file 12879_2022_7846_MOESM5_ESM.docx]

**Additional Methods**

***Data Generating Mechanism***

The model described in Imperial et. al. utilized individual-level data (n = 3405) from three international, randomized phase 3 trials (Ofloxacine-Containing, Short-Course Regimen for the Treatment of Pulmonary Tuberculosis [OFLOTUB] trial, NCT00216385; Rapid Evaluation of Moxifloxacin in TB [REMoxTB] trial, NCT00864383; and High-Dose Rifapentine with Moxifloxacin for Pulmonary Tuberculosis [RIFAQUIN] trial, ISRCTN44153044) that compared 4-month fluoroquinolone-containing regimens to the standard 6-month regimen for treatment of drug-susceptible TB.

The hazard risk for TB-related outcomes was best described with a surge function [EQ1]. A decreased number of treatment days increased the baseline hazard risk (𝜆) of TB-related outcomes (29% [percent relative standard error (%RSE) = 9] increase per 28-day decrease in number of treatment days. Baseline factors that increased hazard risk included HIV coinfection (86% [%RSE = 29] increase), higher smear grade (68% [36] increase for smear 3+ relative to smear 1+ or negative), male sex (64% [32] increase), presence of cavitary disease (26% [57] increase), and lower body mass index (BMI) (18% [41] increase per 5 kg/m2 decrease). Inclusion of time to culture conversion improved discrimination with an increase in ROC AUC from 0.69 (95% CI, 0.66–0.72) to 0.72 (0.69–0.75).

ℎ(𝑡,𝑥𝑖) = 𝜆(𝑥_𝑖_)𝑡^𝛽^exp(-𝛼𝑡) (1)

The hazard risk for culture conversion was also best described with a surge function. There were several baseline factors that increased the baseline hazard risk (𝜆) for culture conversion including: younger age (8.8% [21] increase for every 10 years younger), lower smear grade (162% [24] increase for smear 1+ and 101% [28] increase for smear 2+ relative to smear 3+), clinical site (79% [6] decrease for Asia site and 156% [52] increase for India site relative to sub-Saharan Africa site). Additionally there were several baseline factors that increased the shape hazard risk (β) for culture conversion including: lower smear grade (44% [22] increase for smear 1+ and 36% [26] increase for smear 2+ relative to smear 3+) and clinical site (49% [13] decrease for Asia site and 22% [78] increase for India site relative to sub-Saharan Africa site).

Individual survival probability density functions are calculated for each individual participant for culture conversion using the factors described above plus the regimen hazard ratios as described in Table 1. Then a random number between 0 and 1 is assigned to that participant and where it intersects the probability density function determines the TCC. The regimen hazard ratios, TCC, and aforementioned factors are used to calculate another survival probability density function for TB-related unfavorable outcomes. Another random number is assigned to that participant and the individual participant TTR calculated.

***Bayesian Response Adaptive Randomization***

*Intermediate and Final Endpoints*
The primary endpoint measured is treatment success at 78 weeks (TS-78) after randomization, and is defined as relapse free survival up to 78 weeks post randomization (TTR ≥ 78 weeks). Three intermediate endpoints will be measured at 8 weeks (TS-8: TCC ≤ 8 weeks), 24 weeks (TS-24: TCC ≤ 24 weeks AND TTR ≥ 24 weeks), and 52 weeks (TS-52: TTR ≥ 52 weeks). We expect a strong correlation to be observed between the intermediate endpoints TS-24 and TS-52 with the final endpoint TS-78; therefore, Phase IIc will adaptively randomize based on TS-24 and seamless Phase II/III based on TS-52. Incorporating relapse data into adaptive randomization is feasible for a seamless Phase III trial as the longer recruitment period permits the accumulation of relapse data in time to affect the course of the trial. As the majority of relapses (75-85%)^33^ occur within 6 months post-treatment, TS-52 was chosen as reasonable predictor for treatment outcomes at 78 weeks. Individual patient endpoints were model generated as TCC and TTR then converted into binary TS-8, -24, -52, and -78 and used to update priors as data accumulates.

*Adaptive Randomization*Phase IIc BAR is dependent on the Bayesian probability that TS-24 in arm *k* is better than the control arm. Essentially, as evidence accumulates of arm *k* performing better than the control arm, the randomization probability to arm *k* will be adjusted higher. For the control, the randomization probability is defined so that the control sample size approximately matches the investigational arm with the highest number of enrolled patients. The mathematical framework was drawn from Cellamare et. al. 2016 which implemented a BAR design for the endTB trial. Two randomization tuning parameters *ɣ* and *η*, where *ɣ* tunes how heavily randomization probabilities are weighed in favor of well performing arms and *η* tunes how quickly adaptive randomization responds to incoming data—*η* > 1 weighs early data higher and responds quickly, *η* < 1 penalizes early data and waits for more data before responding to accumulated data. These parameters were explored in a grid-like fashion across a range of reasonable values and optimized for graduating clinically noninferior arms and stopping suboptimal arms.

$$EQ1: P\left( TS-52 \left( k \right) \right)= \psi_{1.1}\left( k \right)ɸ\left( k \right)\omega\left( k \right) + \psi_{0.1}\left( k \right)\left( 1 - ɸ\left( k \right) \right)\omega\left( k \right)+ \psi_{1.0}\left( k \right)ɸ\left( k \right)\left( 1-\omega\left( k \right) \right) + \psi_{0.0}\left( k \right)\left( 1 - ɸ\left( k \right) \right)\left( 1-\omega\left( k \right) \right)$$

The Phase II/III seamless Bayesian design was constructed using the same framework, but modified so that adaptive randomization is dependent on TS-52 instead, which is estimated by [EQ1] where ϕ(*k*) represents the probability of TS-8, ω(*k*) represents the probability of TS-24, and *ψ_i.j_*(*k*) represents the conditional probability of TS-52 given the four combinations of positive or negative TS-8 and TS-24. The priors for TS-8 and TS-24 are uniform distributions, the priors for the TS-52 conditional probabilities are: *ψ_1.1_* = (10,1) where *ψ_1.1_* represents a positive TS-8 and TS-24, *ψ_1.0_* = (1,10) where *ψ_1.0_* represents a positive TS-8 and negative TS-24, *ψ_0.1_* = (10,1), *ψ_0.0_* = (0.1,10). Optimistic (10,1) and skeptical (3,8) priors for *ψ_0.1_* were tested to determine the effects of confidence in the relationship between month 2 culture status and relapse.

A maximum N per arm rule was designed to stop enrollment to arms in which a sufficient number of patients have already been enrolled, 100 patients for phase IIC and 400 for seamless (same maximums as MAMS). Stopping rules for the Bayesian trial designs were designed to graduate a maximum number of arms, 4 for Phase IIc and 3 for seamless Phase II/III, to reflect the desired number of candidates to advance to Phase III or submission to regulatory approval.

**Sensitivity Analyses**

Decoupling the model assumed relationship between individual TCC and TTR did not significantly affect the conclusions of each of the designs. Since MAMS evaluates arms as a whole, graduation and stop decisions were not affected. For BAR however, because the definition of TS-24 (TCC ≤ 24 weeks AND TTR ≥ 24 weeks) relies on an individual’s TCC-TTR relationship, each arm’s TS-24 rate changed slightly. Since the Phase IIc BAR design randomizes based on each arm’s TS-24 rate relative to other arms, adaptive randomization responded appropriately to the decoupled TS-24 profile and shifted patients from desirable to minimal regimens. We believe our conclusions are not affected because the BAR design does not inherently assume an individual’s TCC-TTR relationship, and the observed differences are an artifact of model implementation and the TS-24 definition, not a weakness of the BAR design. In practice, even if the relationship between TCC and TTR is different from what has been observed in prior trials each arm’s TS-24 rate would still remain the same. In support of this conclusion, seamless BAR, which randomizes based on TS-52, remained unaffected.

Changing the composition of regimens changed BAR patient allocation across arms in expected ways. Since adaptive randomization is dependent on each arm’s performance relative to other arms, the BAR design will allocate patients very similarly between a scenario with 4 desirable and 4 minimal regimens and a scenario with 4 minimal and 4 suboptimal regimens. In other words, without additional trial rules a BAR design would allocate resources to the best regimens from a pool of suboptimal candidates and it is therefore not possible to design such a trial that achieves our goals of stopping suboptimal regimens x% of the time when there are few desirable regimens.

We are simulating data for time to relapse from a parametric survival model (based on previous work) and therefore the ‘true relapse rate’ for a particular simulation depends upon the exact individual patients recruited into each arm, in addition to the stochastic survival model, there is no underlying ‘true relapse rate’ for an arm. Therefore, percent bias was assessed using the median relapse rate from unbiased non-adaptive trial simulations (Table 1) as reference, where 2000 patients were recruited into each arm using fixed randomization. Percent bias was calculated for each arm *k* as 100*(pathway relapse rate_k_ – median unbiased relapse rate_k_)/ median unbiased relapse rate_k_.

A long delay in biomarker data results in inefficiency of an adaptive design. We ran simulations comparing the 6 week lag time (used in all simulations in the main text) to an 8 week lag time as a sensitivity analysis. Minimal differences were observed, with graduation and stopping rates changing less than 2% for *desirable* or *suboptimal* regimens and less than 5% for *minimal* regimens.

**Sources of Variation**

Important to consider while interpreting simulation results is the sources of variation. There are two sources of variation in this study: (1) The stochastic nature of parametric survival models. Each individual patient has an individual distribution of potential TCC and TTR based on their individual clinical and disease characteristics. Each patient’s distribution is calculated and sampled upon their recruitment and due to not fixing the random seed, recruiting the same patient multiple times will result in slightly different TCC and TTR. (2) The randomization of patients into arms. Although randomization is balanced so that easy, moderate, and hard-to-treat patients are equally represented in all arms, with enough simulations an arm could be comprised of higher risk patients from each group, thus underestimating the efficacy of said arm.

*Implications of Variation*

Although this variation makes interpreting the simulation results more challenging and nuanced, simulating this variation (instead of assuming a fixed culture conversion rate and relapse rate in each arm as previous studies have done) is reflective of the full range of what may be observed in reality. Interestingly, the variation affects the course of MAMS and BAR trials differently. As the MAMS design compares each regimen to the control independently, the variation in other experimental arms is irrelevant to the comparison at hand. For example, if a suboptimal regimen has a higher survival rate than its true rate due to variation in patient response and randomization, the suboptimal regimen may be graduated but it is irrelevant to the evaluation of the other experimental arms in the trial. However, for a BAR design, since randomization is dependent on the performance of other arms, the variation in a particular arm affects the course of the trial as a whole. Using the same example, if a suboptimal regimen has a higher survival rate than its ‘true’ rate (median cure rate from Table 1), then patient allocation shifts from other arms to the suboptimal regimen. In this scenario for patient enrollment across arms to be the same as the true scenario, all arms would also have to have a proportionally higher survival rate than its true rate.
