# Supplementary material for: A comparison of clinical development pathways to advance tuberculosis regimen development
Source: BMC Infect Dis. 2022 Dec 9;22:920. doi: 10.1186/s12879-022-07846-w (PMC9733404; doi:10.1186/s12879-022-07846-w)
Supplement: Supplementary file 4 — Additional file 4: Figure S4. Comparison of Performance Measures. (A) Estimated relapse rate across arms and trial designs is accurate when compared to unbiased simulations. Lower accuracy and higher 95% prediction intervals are observed in arms with very low sample size, i.e. phase IIC BAR arms 7, 8, 9. (B) The higher number of relapse events observed in BAR designs’ desirable regimens provide greater evidence of efficacy for the best regimens. Given the low relapse rate in better regimens, higher sample size is particularly needed in the best regimens to observe an appreciable number of relapse events to produce an accurate relapse rate estimate. (C) Minimal bias is observed in estimation of relapse rate across designs, except for phase IIC BAR arms 7, 8, 9 where the low sample size produces a consistent underestimation of relapse rate. Overall, both MAMS and BAR designs provides excellent accuracy and precision in relapse rate estimation. The bias in suboptimal regimens is inconsequential as these arms would quickly be abandoned for lack of efficacy. [file 12879_2022_7846_MOESM4_ESM.pdf]

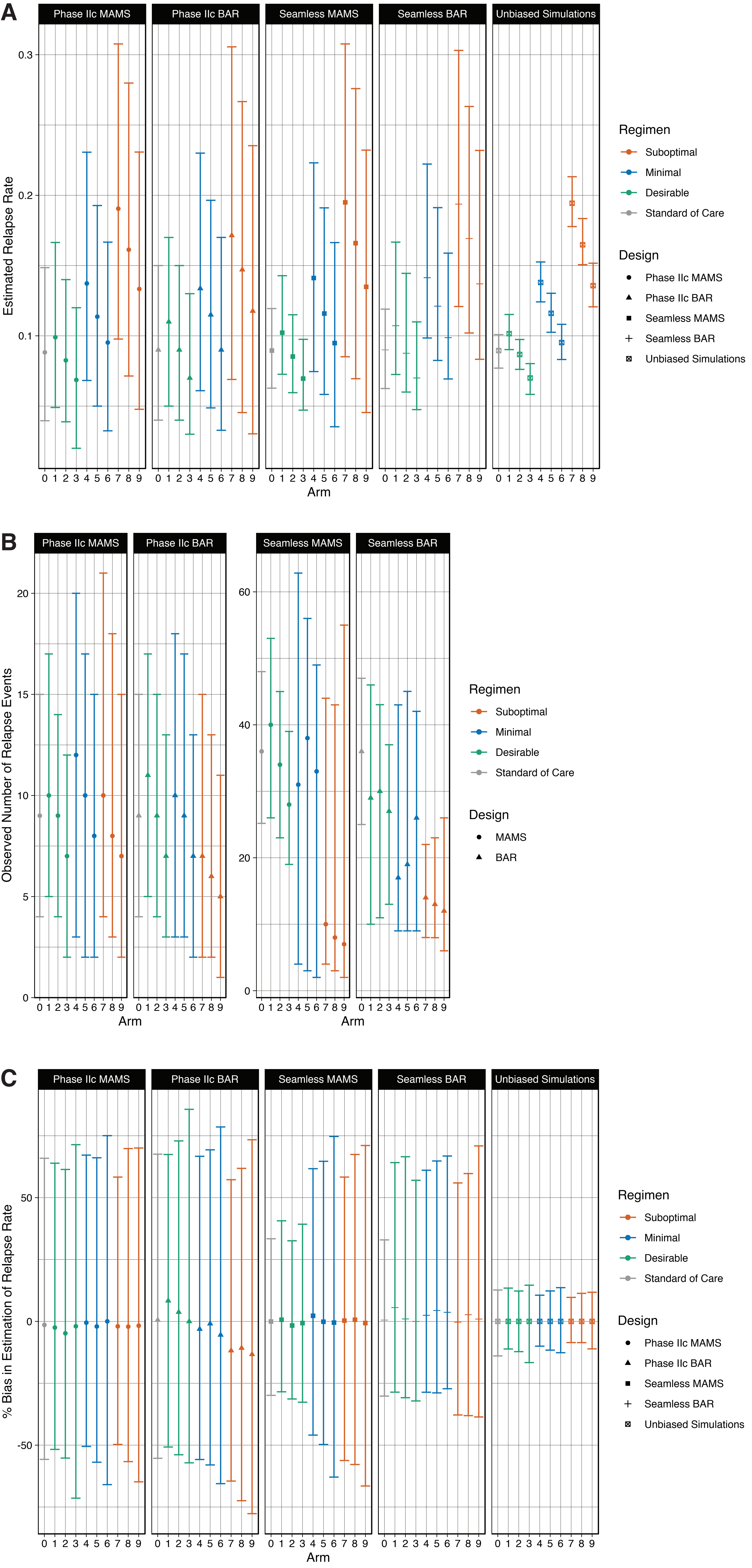
A
Phase IIc MAMS
Phase IIc BAR
Seamless MAMS
Seamless BAR
Unbiased Simulations
Estimated Relapse Rate
Arm
Regimen
Suboptimal
Minimal
Desirable
Standard of Care
Design
Phase IIc MAMS
Phase IIc BAR
Seamless MAMS
Seamless BAR
Unbiased Simulations
B
Observed Number of Relapse Events
Arm
Regimen
Suboptimal
Minimal
Desirable
Standard of Care
Design
MAMS
BAR
C
% Bias in Estimation of Relapse Rate
Arm
